# *Arabidopsis*-Based Dual-Layered Biological Network Analysis Elucidates Fully Modulated Pathways Related to Sugarcane Resistance on Biotrophic Pathogen Infection

**DOI:** 10.3389/fpls.2021.707904

**Published:** 2021-08-19

**Authors:** Hugo V. S. Rody, Luis E. A. Camargo, Silvana Creste, Marie-Anne Van Sluys, Loren H. Rieseberg, Claudia B. Monteiro-Vitorello

**Affiliations:** ^1^Departamento de Genética, Escola Superior de Agricultura Luiz de Queiroz, Universidade de São Paulo, Piracicaba, Brazil; ^2^Centro de Cana, IAC-Apta, Ribeirão Preto, Brazil; ^3^Departamento de Botânica, Instituto de Biociências, Universidade de São Paulo, São Paulo, Brazil; ^4^Department of Botany, University of British Columbia, Vancouver, BC, Canada

**Keywords:** biological networks, data mining, data integration, transcriptome, biotrophic pathogens, *Saccharum*

## Abstract

We assembled a dual-layered biological network to study the roles of resistance gene analogs (RGAs) in the resistance of sugarcane to infection by the biotrophic fungus causing smut disease. Based on sugarcane-*Arabidopsis* orthology, the modeling used metabolic and protein-protein interaction (PPI) data from *Arabidopsis thaliana* (from Kyoto Encyclopedia of Genes and Genomes (KEGG) and BioGRID databases) and plant resistance curated knowledge for Viridiplantae obtained through text mining of the UniProt/SwissProt database. With the network, we integrated functional annotations and transcriptome data from two sugarcane genotypes that differ significantly in resistance to smut and applied a series of analyses to compare the transcriptomes and understand both signal perception and transduction in plant resistance. We show that the smut-resistant sugarcane has a larger arsenal of RGAs encompassing transcriptionally modulated subnetworks with other resistance elements, reaching *hub* proteins of primary metabolism. This approach may benefit molecular breeders in search of markers associated with quantitative resistance to diseases in non-model systems.

## Introduction

Plant defense mechanisms against pathogens are a multi-layered complex of biological interactions. Resistance signaling cascades are triggered in plants through direct and indirect associations of resistance proteins with either pathogen/microbe/damage-associated molecular patterns (PAMP, MAMP, and DAMP) or more target-specific effector proteins (Jones and Dangl, 2006; Macho and Zipfel, 2014). Other mechanisms such as the guardee hypothesis (Dangl and Jones, 2001; Jones and Dangl, 2006), the decoy model (Van Der Hoorn and Kamoun, 2008), and the formation of multi-protein R-complexes (Friedman and Baker, 2007) have also been shown to trigger resistance in plants. Furthermore, quantitative disease resistance (QDR) is predominant in crops and is conferred by a complex association of multiple plant pathways (Delplace et al., 2020).

Resistance (R) genes harbor conserved features frequently used in genomic studies searching for associations between RGAs and disease resistance in crops (Hübner et al., 2019; Neupane et al., 2019; Rody et al., 2019). Despite the wealth of available “omics” data, understanding the dynamics of biochemical interactions within and across organisms remains challenging. Network analysis based on graph theory is a critical component of systems biology and offers a promising approach for reconstructing complex biological networks and integrating omics with primary biological data (Junker and Schreiber, 2008; Peyraud et al., 2017). Although promising in humans (Khorsand et al., 2020; Kösesoy et al., 2021) and *Arabidopsis* (Li et al., 2017), the modeling and analysis of biological networks have yet to be widely applied to study plant-pathogen interactions of non-model organisms such as sugarcane.

Sugarcane is the primary crop of the world for sugar and biofuel production. Despite its economic importance, no large-scale databases such as KEGG (Kanehisa et al., 2004) and BioGRID (Oughtred et al., 2019) provide interaction information for sugarcane. Due to the complex combination of polyploidy and aneuploidy (D’Hont et al., 1998; Piperidis and D’Hont, 2020), only recently has an allele-defined genome sequence, one of the principal ancestors of modern sugarcane, the *Saccharum spontaneum* clone AP85-441, become available (Zhang et al., 2018). Subsequently, a monoploid version of the modern sugarcane R570 (Garsmeur et al., 2018) and a gene space assembly of the SP80-3280 genotype (Souza et al., 2019) have contributed further to start a new genomic era for sugarcane breeding.

In this study, we modeled a dual-layered (metabolic and PPI layers) biological network for sugarcane using interaction data from *Arabidopsis thaliana* and curated interaction knowledge regarding plant resistance for Viridiplantae obtained through text-mining of the UniProt/SwissProt database (Boutet et al., 2007). We integrated multiple functional annotations and transcriptome data (Rody et al., 2019) from two sugarcane genotypes exhibiting different levels of resistance to biotrophic *Sporisorium scitamineum*, the causative agent of smut disease. Then, we investigated whether the model would help us understand the roles of RGAs and subsequent molecular events following biotrophic pathogen infection. Both genotypes had subnetworks encompassing fully transcriptionally modulated paths from the peripheral RGAs to the central *hub* proteins of primary metabolism. We show that differences in the arsenal of expressed RGAs between the two genotypes are potentially related to chromosome ancestry, offering clues regarding the ultimate source of augmented pathogen perception by the resistant genotype.

## Results

### The Dual-Layered Network

A total of 9,186 nodes (proteins and/or reactions) and 42,499 edges (interactions) formed the multigraph of the *Arabidopsis*-based dual-layered sugarcane network, harboring 428 connected components in a disconnected network. The largest connected component of the network (Table 1) was comprised of 8,548 (93%) nodes and 42,243 (99%) edges. Because (1) some statistics require the network to be fully connected to be applied and (2) finding pathways was the main focus of this study, we considered the latter in all downstream analyses.

**TABLE 1:** Dual-layered network statistics.

Statistics	Value
Diameter length	29
Connectivity density	0.0011
Average shortest path length (ASPL)	5.39
Average clustering coefficient (ACC)	0.31
Cutting vertices protein nodes	654

**FIGURE 1:**
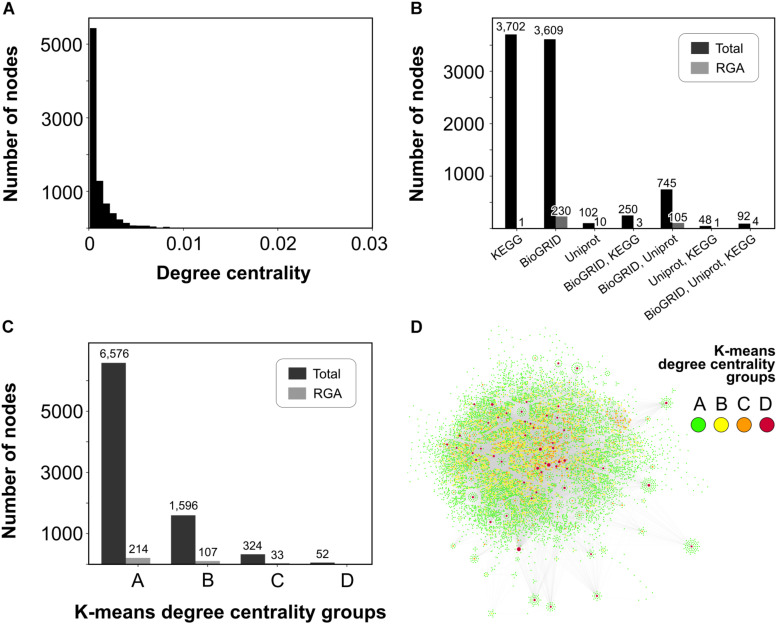
Overview of the sugarcane dual-layered network topology. **(A)** Histogram of degree centrality showing the majority of nodes with low values. **(B)** The number of nodes from each of the three interaction databases is used in this work. **(C)** The number of total nodes and nodes harboring RGA orthologs in each of the four *K*-means degree centrality groups of **(A)** less connected, **(B)** intermediate connected, **(C)** highly connected, and **(D)** super-connected nodes. **(D)** Graph view of the largest component of the sugarcane dual-layered network with nodes colored according to the legend.

The sugarcane dual-layered network exhibited a highly inhomogeneous and long-tailed degree distribution (Figure 1A), with many nodes forming few connections, and few nodes forming many connections. Likewise, the K-means unsupervised learning algorithm predicted that most nodes (*N* = 6,576) belong to group A of less connected nodes (Figure 1C), and only a few super-connected nodes belong to group D (*N* = 52) (Figures 1C,D). Nodes within-group D were also considered *hubs*.

Results from the Molecular Complex Detection (MCODE) algorithm also illustrate the inherent modularity of biological networks. MCODE predicted 3,139 nodes within 341 different densely connected modules. Overall, there was a consistent overlap of nodes from the three different interaction datasets (KEGG, BioGRID, and SwissProt) (Figure 1B). The PPI layer had 4,696 nodes connected to 2,187 first neighbor nodes from the metabolic layer, which allowed us to explore signaling transduction on pathogen infection through both metabolic reactions (enzyme catalysis) and protein-protein interactions (PPIs). Most of the nodes and interactions predicted using text mining were also predicted by either BioGRID or KEGG databases (Figure 1B). Only 102 nodes (1.19% of 8,548 nodes), including 10 RGA nodes, were predicted using text mining over UniProt/SwissProt.

### RGAs Nodes Within the Dual-Layered Network

We supplied information from an annotated set of sugarcane RGAs (Rody et al., 2019) into the network. Most of the RGA nodes (nodes having at least one sugarcane RGA ortholog) were within the less connected group A (*N* = 214) (Figure 1C), and therefore, RGA nodes were mainly at the periphery of the dual-layered network. No RGA nodes were within the super-connected nodes of group D. PPI layer (*N* = 342) and text mining annotations (*N* = 120) predicted most of the RGA nodes, with an overlap of 109 nodes. Only nine RGA nodes were within the metabolic layer (Figure 1B).

We identified 654 protein nodes (excluding reaction nodes) as cutting vertices (CV) in the dual-layered network (Supplementary Table 1). It is noteworthy that 37 RGA nodes were among CV protein nodes (RGA-CV nodes) (Table 2). From these, eight RGA-CV nodes were classified within the highly connected centrality group C, including orthologs of the well-studied leucine-rich repeats (LRR) proteins of BAK1, BRI1, FLS2, and BRL2 regulators of the immune response in *Arabidopsis*.

**TABLE 2:** The number of RGA nodes identified as the CV in three centrality groups of the dual-layered network.

Centrality group	Number of RGA CV nodes	Number of sugarcane orthologs	Nodes
A	7	45	AHK3, CESA6, AT4G21380, AT2G42620, AT2G45140, AT4G23180, AT1G04960
B	22	126	EFR, COI1, AB36G, AHK4, CESA3, CEPR2, CLV1, AHK2, MIK1, PXL1, 2.4.1.43, AT3G56370, AT5G56750, AT5G64560, AT3G05710, AT3G24660, AT5G47910, AT4G34220, AT5G67280, AT4G04570, AT5G49770, AT4G23270
C	8	61	BAK1, FLS2, BRI1, Y5838, SUVH2, BRL2, AT5G46860, AT1G45145

*CV, cutting vertices; RGA, resistance gene analogs.*

### RGAs in Core-Periphery Strongly Connected Components

We interrogated the network for information about the roles of differentially expressed RGAs (RGA-DEs) using data from previous experiments with susceptible (IAC66-6) and resistant (SP80-3280) sugarcane genotypes inoculated with smut biotrophic pathogen. By applying the expression values of the sugarcane ortholog with the smallest DE *p*-value as attributes of nodes, we investigated whether the RGA-DE nodes were within the predicted MCODE modules and which other DEGs were within the same modules.

**FIGURE 2:**
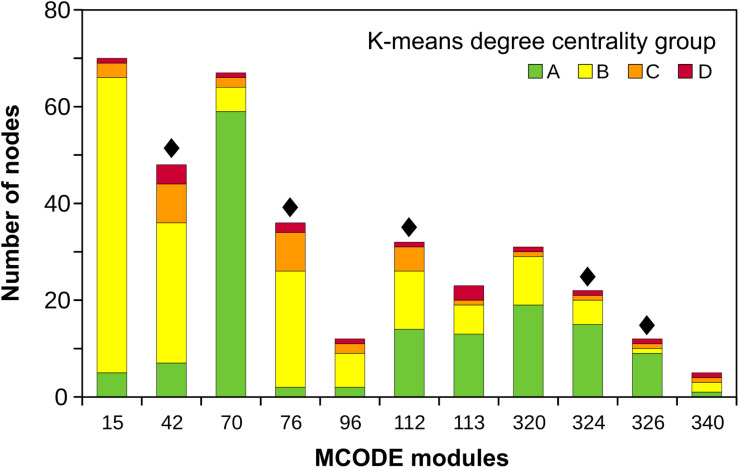
Eleven MCODE core-periphery modules harboring nodes from all the four *K*-means degree centrality groups colored according to the legend. Black diamonds indicate modules harboring RGA nodes.

Eleven MCODE modules (Supplementary Table 2) covered nodes extending from the core to the outermost peripheral nodes (A to D centrality groups) (Figure 2). Of these, five modules included RGA nodes (Figure 2; indicated with black diamonds), and three modules had RGA-DE nodes: MCODE 42 (6 RGA-DE nodes from IAC and 10 from SP experiments), MCODE 76 (1 from IAC and 6 from SP), and MCODE 324 (2 from IAC). These three modules matched the pattern we were seeking; they were core-periphery and included DEGs and RGA-DEs. Focusing on the core-periphery modules, we could infer if RGAs participated in transcriptionally modulated pathways and offer clues about other proteins that might influence the overall response.

**FIGURE 3:**
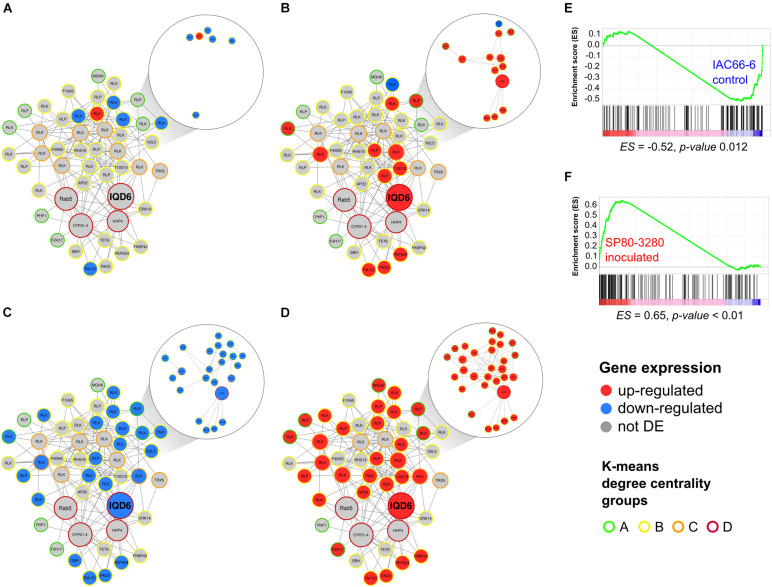
Graph overview of core-periphery MCODE module 42 (48 nodes and 166 edges) alongside the expression profiles of genes within nodes from two sugarcane transcriptome experiments of IAC66-6 and SP80-3280. **(A)** IAC DEGs, **(B)** SP DEGs, **(C)** IAC GSEA leading-edge genes, **(D)** SP GSEA leading-edge genes. Node sizes represent centrality degree values. Node expression profiles are colored according to the legend, standing for not differentially expressed (DE), upregulated or downregulated, relative to sugarcane orthologs with the lowest *p*-value of each node in the two sugarcane transcriptome experiments. Node edges are colored according to the legend of the *K*-means degree centrality groups. Edges connecting DE nodes were also colored as gray, red, or blue, indicating the expression profile pattern of the node. GSEA signatures of IAC and SP in **(E)** and **(F)**, respectively, show the module 42 gene set distribution.

The MCODE module 42 was enriched with orthologs annotated as from the KOG T category of signaling transduction and showed the greatest difference between the expression profiles of IAC and SP within nodes (Figure 3). It included 48 nodes, and of these, 33 were RGA nodes. Most of the RGA orthologs in the nodes of MCODE 42 were from the TM-LRR family (RLPs and RLKs), and one node had an RGA ortholog from the TM-CC class. Besides RGAs, module MCODE 42 included nodes containing orthologs of the WD40-like repeat protein (*N* = 1), the RAF-like subtype of the MAPKKK family (*N* = 2), proteins related to cell wall/membrane functions (*N* = 8), and transport (*N* = 15), and proteins related to oxidative burst (*N* = 5) (Supplementary Table 3). Four of these nodes belonged to the super-connected group D (Figure 3), which included orthologs of IQ-domain 6 (IQD6, AT2G26180), cyclophilin-like peptidyl-prolyl *cis-trans* isomerase family (AT3G66654), Rab5-interacting family (AT5G49540), and heptahelical protein 4 (AT4G37680). They all had orthologs in the sugarcane database (Supplementary Table 3).

The IQD6 was the only CV node from the MCODE 42 module found within the centrality group D (and between the centrality group C). Consequently, the IQD6 node was considered of high importance in that module according to two centrality measures, which placed it as highly central in the network and situated in the path to connect a high number of other nodes. Importantly, IQD6 was connected to upregulated nodes in which the edges cross-less connected nodes, reaching more peripheral RGA-DE nodes (Figure 3B).

Most of the IAC RGA-DE nodes were downregulated (5 downregulated and 1 upregulated) in the MCODE 42. Only one RGA, an ortholog of *Arabidopsis* PSKR1, was identified as upregulated. On the contrary, SP had 10 RGA-DE nodes in MCODE 42 (1 downregulated and 9 upregulated).

Besides using DE data, we applied the computation method of Gene Set Enrichment Analysis (GSEA) to identify gene set enrichment in the transcriptome data. GSEA also showed widely divergent expression signatures between IAC and SP transcriptomes (Figures 3C,D). Distribution plots showed distinct peaks at the end for IAC (Figure 3E and Supplementary Table 4) and at the beginning for SP (Figure 3F and Supplementary Table 5) of the GSEA ranked gene list, indicating a subset of members that contributed most to the enrichment score (ES). Because we used comparisons of inoculated vs. control transcriptome experiments, the positive value of ES for SP (*p* < 0.01) suggested a positive correlation with the inoculated treatment, whereas the negative ES value of IAC (*p* < 0.05) was consistent with the negative regulation in the inoculated treatment. Leading-edge genes in the SP included two IQD6 orthologs, and several TM-LRR RGAs (*N* = 25), orthologs of the *STRUBBELIG*-receptor family (*N* = 13), fatty alcohol oxidase (*N* = 2), and respiratory burst oxidase homolog D (*N* = 3). There were 56 leading-edge genes in the SP experiment (Figure 3F), whereas 42 leading-edge genes were detected in the IAC experiment (Figure 3E). Not previously identified by DE analysis, GSEA showed that the super-connected *hub* IQD6 had a modulated path to peripheral RGA-DE nodes in both IAC and SP experiments (Figures 3A–D).

### Core-Periphery Subnetworks: From RGAs to the Core of Metabolism

We also investigated if RGA-DEs participated in fully transcriptionally modulated core-periphery sub-networks other than MCODE modules. To achieve this purpose, we used the depth-first search (DFS) algorithm with the RGA-DE nodes as sources and traversed the network across other DEGs. We found one fully modulated core-periphery sub-network for the SP (SP2) and two for the IAC (IAC1 and IAC9) transcriptome (Supplementary Table 6).

There were similarities among the largest sub-networks identified in SP (SP2, *N* = 332 nodes) and IAC (IAC9, *N* = 108 nodes). The highest percentage of node matches occurred in the centrality group D (41.67%) and decreased progressively in the less connected groups, reaching the smallest node match percentage in group A (2.76%) (Table 3). RGAs represented the major divergence between the IAC9 and SP2.

**TABLE 3:** Comparison by *K*-means degree centrality groups between the core-periphery sub-networks of IAC9 and SP2 generated through the depth-first search (DFS) of the RGA-DE nodes from transcriptome experiments with IAC and SP.

Centrality group	Total nodes	IAC9 nodes	SP2 nodes	Matches	Mismatches	Match%
**All nodes**						
A	181	26	160	5	176	2.76
B	146	48	123	25	121	17.12
C	52	28	38	14	38	26.92
D	12	6	11	5	7	41.67
**RGA-DE nodes**						
A	18	1	17	0	18	0.0
B	24	2	24	2	22	8.33
C	7	1	7	1	6	14.29

*RGA-DE, differentially expressed RGAs.*

Despite the elevated (∼41%) node matches in group D, the expression profile of those matching nodes also diverged, to some extent, between transcriptomes. Examples are node 2.7.1.40 of pyruvate kinase (IAC upregulated; SP downregulated) and the AT4G26630 node corresponding to a DEK-domain containing protein 3 (DEK3), which is involved in chromatin remodeling (IAC downregulated; SP upregulated). Although not further analyzed in this study, other nodes related to the glycolysis pathway were also identified as harboring DE sugarcane orthologs (Supplementary Figure 1).

### Genomic Features of RGA-DEs Found in Core-Periphery Subnetworks

**FIGURE 4:**
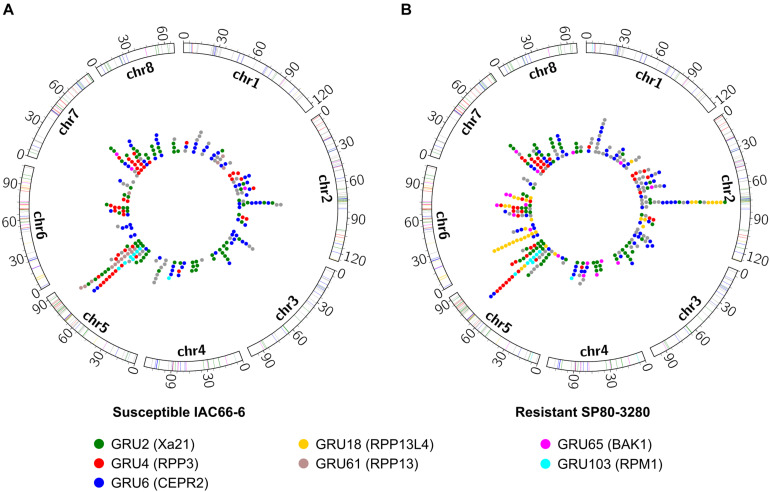
Distribution of *Saccharum spontaneum* orthologs of RGAs identified as within core-periphery subnetworks and DE in each of the two sugarcane transcriptome experiments of **(A)** susceptible IAC66-6 genotype and **(B)** resistant SP08-3280 genotype. Colored dots within circles indicate the positions of RGA orthologs along eight chromosomes, as well as the colored traces in chromosome bars. Colors depict seven main orthogroups according to the legend, and gray color depicts orthologs from other orthogroups.

*Saccharum spontaneum* (AP85-441) orthologs found within the same orthogroups as the sugarcane RGA-DEs present in core-periphery subnetworks were investigated for their clustering and genome organizations. A total of 82 RGA-DEs were unveiled from IAC (*N* = 30) and SP (*N* = 58) core-periphery subnetworks (Supplementary Table 7), delivering a total of 369 ortholog sequences from *S. spontaneum* (Supplementary Table 8). Chromosomes 2, 5, and 6 showed an elevated number of RGA-DE orthologs compared with the other chromosomes, which together harbored 55.8% (*N* = 206) of orthologs (Figure 4). Many RGA-DE orthologs were identified within predicted clusters across the aforementioned chromosomes (Supplementary Figure 2), with an important discrepancy between the IAC and SP RGA-DE orthologs: chromosome 5 had more orthologs of IAC organized in clusters (*N* = 4 IAC; *N* = 2 SP), whereas on chromosomes 2 (*N* = 1 IAC; *N* = 3 SP) and 6 (*N* = 3 SP), there were more ortholog clusters of SP.

Orthologs of SP RGA-DEs, especially RLKs, were more abundant than those of IAC (Figure 4B and Supplementary Table 7), leading to a very distinct chromosomal distribution of orthologs between the IAC and SP experiments. Susceptible IAC had more RGA-DEs orthologs from CNL and TM-CC classes on chromosome 5 than had the SP (Figure 4A). However, resistant SP had more RGA-DEs orthologs from CN and CNL classes (RPM1-like, orthogroups GRU18 and GRU103) on chromosome 6 (Figure 4B). Orthogroups GRU18 and GRU103 represented an important disparity observed among orthologs originating from IAC and SP. Only SP had an RGA-DE predicted as belonging to GRU18. The distribution of orthologs from GRU18 and GRU103 across *S. spontaneum* chromosomes was very dissimilar: GRU18 orthologs were mainly on chromosome 6 (*N* = 20), whereas GRU103 orthologs were mainly located on chromosome 5 (*N* = 9).

Chromosome 6 of *S. spontaneum* also harbored the longest segment of exclusive SP RGA-DE orthologs, including CNLs from GRU18. Another important discrepancy is that both IAC (*N* = 4) and SP (*N* = 2) had orthologs organized into clusters on chromosome 5, whereas only SP (*N* = 3) had orthologs in clusters on chromosome 6 (Supplementary Figure 2). Chromosome 6 of SP also contained a handful of other exclusive RGA orthologs, such as BAK1 (GRU65) and the cellulose synthase A (GRU15). Other RLKs such as Xa21 (GRU2), RPP3 (GRU4), and CEPR2 (GRU6) were shared by the SP and IAC genotypes across different chromosomes (Supplementary Table 8).

## Discussion

Using graph theory, we modeled a dual-layered biological network, at steady state, for the non-model organism sugarcane, based on the metabolic and PPI data for *A. thaliana*. The network allowed us to integrate multiple functional annotations related to the plant immune system and the differential expression (DE) profiles of two sugarcane genotypes having distinct degrees of resistance to the biotrophic fungus *S. scitamineum*. By applying network topology analysis, we demonstrated that the more extensive RGA fighting arsenal of smut-resistant sugarcane is involved in an augmented and fully modulated signaling network that reaches *hub* proteins in the core metabolism. Further, we show that discrepancies in the expression patterns of RGAs between genotypes are potentially related to their clustered arrangement as observed for its orthologs, especially on chromosomes 2 and 6 of *S. spontaneum*.

### The Modularity of Sugarcane Dual-Layered Network

Biological networks exhibit specific organizing principles that reflect the interactions occurring among molecules in the cell(s) of all life domains. Although the assembled sugarcane dual-layered network did not show a degree distribution (Figure 1A) that followed the conceptual power-law pattern in a strict statistical sense, the inhomogeneous and long-tailed distribution suggested an elevated number of nodes performing only a few connections as compared to nodes of high degree values. Consistent with this prediction, only a few nodes were classified into the highly connected C (*N* = 324) or super-connected D (*N* = 52) centrality groups using the unsupervised learning K-means algorithm.

The calculated average clustering coefficient (ACC) of the sugarcane dual-layered network (0.31) was lower than the ACC of the sugarcane metabolic layer alone (0.55) and also lower than the average ACC (0.45; σ 0.0019) of metabolic networks for 95 plant species using KEGG data (data not shown). As a measure of the tendency of nodes to form clusters (Ravasz, 2009), these ACC values indicate that the addition of the PPI layer decreased the network modularity value due to the introduction of many and low connected nodes. Ravasz et al. (2002) observed that the clustering coefficient of metabolic networks was independent of their size among 43 distinct organisms from archaea, bacteria, and eukaryote domains of life. The calculated ACC for metabolic networks of 95 plants followed the observations of Ravasz et al. (2002), which indicates that plants might share similar modularization of their metabolic networks. Most of the nodes had small clustering coefficients in the PPI layer, which led to the calculated ACC equal to 0.10. Although the calculated ACC for the PPI layer alone was similar to what has already been shown in other organisms such as *Saccharomyces cerevisiae* (Uetz et al., 2000), the ACC of plants PPI networks established until now varies largely, from 0.06 in *A. thaliana* to 0.35 in *Oryza* (Chen et al., 2020). Nevertheless, it is known that PPI networks may not reflect the total interactome of an organism due to experimental limitations and that partial sampling can lower the clustering coefficient of networks (Friedel and Zimmer, 2006).

The diameter of the sugarcane dual-layered network (length 29) was comparable with an interactome predicted for *Brassica rapa* (length 32) using *A. thaliana* PPI data (Yang et al., 2013). The average shortest path length of the dual-layered network (5.39), which can be interpreted as another representation of the diameter of a network, was also similar to the calculations of 43 organisms obtained by Jeong et al. (2000). The dual-layered network density was very low (0.001) and comparable with the density value of a calculated *A. thaliana* PPI network (Chen et al., 2020). Biological networks are expected to show sparse connection features, which means that the number of edges tends to be much smaller than that of the number of possible edges. Generally, sparsely connected networks are thought to confer an evolutionary advantage because of the preservation of robustness (Leclerc, 2008). Looking at the densities of the two layers separately, the sugarcane PPI layer was sparser (density of 0.001) than the metabolic layer (density of 0.003), which may be due to the aforementioned limitations of PPI networks.

### Smut-Resistant Sugarcane Harbors an Amplified RGA Signaling

To extend the understanding of the roles of RGAs during the interaction of sugarcane with smut, we researched the topological features of nodes of the dual-layered network housing orthologs of those predicted RGA-DEs. We considered as expression signals of the nodes the profile of the RGA-DE having the lowest *p*-value. Then, (1) identifying densely connected components and (2) traversing the network having RGA-DEs as sources, and only across other DE nodes, we identified fully modulated subnetworks containing nodes from all four centrality groups (core-periphery). Densely connected modules in biological networks are believed to be formed among nodes working toward a shared biological goal (Bader and Hogue, 2003). One core-periphery densely connected module, named MCODE 42, was of particular interest because it showed an elevated number of RGA-DEs (upregulated) in the SP experiment as compared to IAC, mostly annotated for the signal transduction KOG category. Further, only in SP did the peripheral RGA-DE nodes have a path across other DE nodes (mostly upregulated) to reach the DE *hub* IQD6 node. These results are consistent with the formation of a larger and modulated subnetwork among the DE nodes (RGAs or not) in the SP experiment, but not in IAC.

Within MCODE 42, GSEA uncovered more genes contributing to the distinct expression profiles of inoculated and control experiments of the two sugarcane genotypes. A goal of GSEA is to provide a more robust way to compare independently derived gene expression datasets (Swarnkar et al., 2015). In the IAC, GSEA identified a negative correlation of leading-edge genes after smut inoculation, whereas a positive correlation was observed in the SP with the inoculation of correspondent leading-edge genes. The *hub* IQD6 ranked as highly important for MCODE 42, was among the antagonistic correlations with inoculated IAC (negative) and SP (positive). Bürstenbinder et al. (2017) suggested IQDs as hubs in cellular auxin and calcium signaling, ultimately regulating plant growth and development. A comprehensive characterization performed on IQD members of *A. thaliana* and *Phyllostachys edulis* suggested their roles in assembling macromolecular complexes orchestrating Ca^2+^ CaM signaling from the membrane to the nucleus (Wu et al., 2016; Bürstenbinder et al., 2017).

Traversing the dual-layered network from RGA-DE nodes and only across DEGs unraveled the two genotypes having subnetworks with the highest content of node matches (about 41%, 5 nodes) among the super-connected nodes (IAC9 and SP2) (Table 3). Pathogens from different kingdoms have been found to deploy an apparatus of virulence proteins to interact with a limited set of highly connected cellular *hubs* and ultimately facilitate infection (Mukhtar et al., 2011). Despite similarities, the two genotypes had distinct expression profiles of some super-connected nodes/proteins from the glycolysis pathway, for example. Whether these changes are related to the demands of energy or signaling for defense mechanisms remains to be further explored (Katagiri, 2004; Bolton, 2009; Shulaev et al., 2011).

Importantly, the smut-resistant sugarcane presented an augmented and divergent arsenal of resistance peripheral nodes (A and B groups) having a fully modulated path to the core of plant metabolism. With the major divergence occurring among the RGA-DE content of genotypes, divergent DEGs also included orthologs involved in signal transduction, MAPKs, cell wall and membrane functions, transport, and proteins related to oxidative burst. Previously, we demonstrated that smut-resistant sugarcane plants activated an early basal defense (48 HAI) involving the oxidative burst (Peters et al., 2017; Rody et al., 2019).

### Amplified RGA Signaling May Be Due to Clustering Organization in a Few Chromosomic Regions

Disease resistance of modern sugarcane cultivars is derived from the 10–15% of chromosomes inherited from *S. spontaneum* (Zhang et al., 2018; Piperidis and D’Hont, 2020). Nucleotide-binding site encoding genes, related to disease resistance, are mostly located on four rearranged chromosomes (2, 5, 6, and 7) of *S. spontaneum* (Zhang et al., 2018; Rody et al., 2019), and up to 39% of sugarcane RGAs have been demonstrated to be organized in clusters (Rody et al., 2019).

We now demonstrate that despite the increased numbers on chromosome 5 of RGA-DEs orthologs from both IAC and SP, the other *S. spontaneum* rearranged chromosomes 2 and 6 (2 and 7 in *S. bicolor*) contained a predominance of RGA-DE orthologs exclusively found in the core-periphery subnetworks obtained with the RGA-DEs from SP (Figure 4). Whereas RLKs largely account for the larger number of smut-responsive RGAs of the SP genotype, a set of CN (*N* = 5) and CNLs (*N* = 9) RGA-DE orthologs localized on chromosome 6 were now uncovered as exclusive to SP (Supplementary Table 8). These differences in the chromosomal composition of RGA-DE orthologs between the two transcriptomes appear to relate to their breeding history. The IAC66-6 line is derived from a cross between Co419 and Co350, which has recent ancestry from *Sorghum durum*, whereas the SP80-3280 is derived from the cross between SP71-1088 and H57-5028 (IAC Sugarcane Breeding Program Databank, Caiana). The recent assembly of 373 k gene spaces of the SP80-3280 genome, alongside predictions of potential transcription factors (TFs) and transcriptional factors binding sites (TFBS) regulatory regions, showed that this genotype harbors a vast repertoire of regulatory elements (Souza et al., 2019). Pathogen elicitors may activate multiple TFs that could ultimately trigger signaling from many defense genes (Zhu et al., 1996; Yang et al., 1997). However, the identification of SP-derived RGA-DE orthologs organized in multiple clusters along the chromosomes of *S. spontaneum* – especially chromosomes 2 and 6 – reinforces the importance of RGAs organization in clusters and might indicate that smut resistance contributes to signaling cascades as a consequence of coordinated expression of RGAs organized in long chromosomal segments, rather than the effect of multiple regulatory regions that may have been lost in the susceptible genomes.

Modern sugarcane cultivars were already shown to host complete versions of *S. spontaneum* chromosomes 2 and 6, and 6–10% of chromosomes originated from interspecific exchanges (Piperidis and D’Hont, 2020). Further, their study supported the hypothesis that modern cultivars predominantly maintain chromosomes of *S. spontaneum* cytotypes with *x* = 8. Modern sugarcanes may also have larger RGA clusters not previously identified in Rody et al. (2019). The average clustering size of AP85-441 was 0.21 Mbp. Haplotype blocks of 1–100 Mbp in size, possibly representing introgressions, were recently shown as associated with relevant traits such as flowering time and dune adaptation of wild sunflowers (Todesco et al., 2020). Although the ancestral *S. spontaneum* has been considered susceptible to smut (Cheavegatti-Gianotto et al., 2011; Silva, 2017), wild accessions collected in Japan were recently found to be resistant (Sakaigaichi et al., 2019).

Further investigations covering RGAs and genomic architecture of smut-susceptible and smut-resistant sugarcane genotypes represent productive approaches for untangling the mechanisms of biotrophic disease progression. The continuous range of smut symptom variation is typical of a quantitative resistance mechanism, and the basal defense is most likely the main response (Niks et al., 2015). Smut-resistance determinants are under constant investigation, and heritability is estimated as moderate (Chao, 1990). Sugarcane is not a model plant, experimentation is demanding, and unlike other crops, near-isogenic lines are hard to obtain due to the nature of the sugarcane hybrid and highly polyploid genome. The biological network proposed in this study offered a feasible alternative to analyzing molecular events and selecting candidates related to resistance not only to sugarcane pathosystems but also potentially to other plant-pathogen interactions. Although the limitations concerning orthology predictions between species that diverged at the basis of flowering plants such as sugarcane and *Arabidopsis*, the latter represents the model organism having the most functional annotation regarding plant-pathogen interactions. In both the metabolic and PPI layers, interactions occurring in *Arabidopsis* may not occur in sugarcane. However, the inherent organization of the core metabolism is conserved among organisms of all three domains of life (Jeong et al., 2000), although there are variations in the constituents of the pathways. As far as we are aware, this is the first large-scale network assembled for sugarcane, and we believe the approach could be broadly applied to other crops and their improvement.

## Materials and Methods

### Genomic and Transcriptomic Data Collection

We obtained a dataset of sugarcane ORFs from the Sugarcane Orthologs of Resistance Database (SORD) (Rody et al., 2019), which contain predictions for a set of 72,269 unique *de novo* transcripts from six sugarcane genotypes (Cardoso-Silva et al., 2014) alongside 16,219 *de novo* assembled transcript sequences from variety RB925345 (Schaker et al., 2016). From SORD, we also obtained a set of 2,470 RGAs sequence predictions. *A. thaliana* protein sequences were obtained from TAIR11^1^. The sugarcane genomic reference of *S. spontaneum* AP85-441 (Zhang et al., 2018) was also recovered. Transcriptome data comprising DE for two sugarcane genotypes having distinct degrees of resistance to smut (IAC66-6 susceptible and SP80-3280 resistant) were obtained from BioProject under accession number PRJNA546134 (Rody et al., 2019). Both transcriptomes were generated for control mock and pathogen-inoculated plants 48 h after inoculation (HAI). For brevity, these two focal transcriptomes are referred to as IAC and SP in the following sections of this study.

### Sugarcane vs. *A. thaliana* Orthologs Predictions

The largest ORF sequences of the sugarcane were used as queries during BLASTp (Altschul et al., 1990) searches against a database assembled with *A. thaliana* protein sequences using a cutoff of e^–05^, minimum of 40% of identity, and 80% of query coverage (parameter-outfmt “6 std qcovs”). For each sugarcane ORF having matches to *A. thaliana* that passed the filter, we defined the best-hit ortholog by ranking the matches by the bitscore parameter. Although BLASTp alone may produce higher error rates than other methods to infer orthology, we considered this method to fit better to the sugarcane sequences and for this study. Besides the phylogenetic distance between sugarcane and *Arabidopsis*, the largest ORFs of the sugarcane were obtained from *de novo* transcripts and may contain partial sequences, impairing the prediction of true orthologs due to assembly biases.

### UniProt/SwissProt Text Mining Resistance-Related Interaction Data

Manually annotated proteins from the structured database of SwissProt (Boutet et al., 2007) were queried through automated Python3 scripts on the UniProt website^2^ using the text terms “plant defense” and “plant resistance.” The retrieved sequence identifiers were downloaded in TXT format, containing all the available annotations, to assemble an initial dataset DatasetTemp (*N* = 8,454). Python3 scripts were then used to parse the DatasetTemp, filter results taxonomically for Viridiplantae, and use regular expression syntax to search for general abbreviations/acronyms (e.g., BAK1 and XA21) that could represent the names of genes/proteins in the following SwissProt annotations such as function, catalytic activity, induction, activity regulation, interaction, and pathway. Significant hits followed by the correspondent species code were initially classified as “putative interactors.” Abbreviations that could not be retrieved from UniProt were discarded as “false putative interactors,” whereas those that could be retrieved were classified as “true putative interactors.” This last step was carried out recursively until no more true putative interactors were retrieved. The final text mining curated knowledge interaction database from SwissProt (*N* = 3,142) was assembled as a tab-delimited file with the following columns: (A) UniProt ID, (B) Interactors ID, (C) EC number(s), and (D) UniProt function. Finally, we performed a new round of BLASTp searches to establish orthology among the retrieved true putative interactors and sugarcane ORFs, using the same approach as used between sugarcane–*Arabidopsis* described in the preceding section.

### Modeling of a Dual-Layered Network

We modeled an *Arabidopsis*-based sugarcane dual-layered network having two layers of interactions among nodes representing proteins. The first layer comprised a metabolic network composed of product-substrate interactions among nodes based on biochemical data of the KEGG database (Kanehisa et al., 2004). The second layer was a PPI network based on physical direct interaction experimental data obtained from BioGRID (Oughtred et al., 2019). Both metabolic and PPI data were obtained for *A. thaliana* and used if there were corresponding ortholog(s) in sugarcane. In addition, a curated knowledge interaction SwissProt database was used. Nodes from different sources and sharing orthologs were merged. All functional annotations obtained were integrated into the network as node attributes: (1) transcript expression data from two different sugarcane experiments (e.g., IAC66-6 48 HAI and SP80-3280 48 HAI), (2) RGA annotation, and (3) functional annotations obtained from the aforementioned interaction databases.

### Modeling of the Metabolic Interaction Layer

Python3 scripts were used to deploy the Bio.KEGG module and the KEGG API pathways data^3^ for the model organism *A. thaliana* to first assemble a local dataset containing the following: (a) Enzyme code (EC) or reaction code, (b) *A. thaliana* gene(s), (c) Reaction codes, (d) Pathway codes, (e) substrates attributed to reactions, and (f) products attributed to reactions. The NetworkX package (Hagberg et al., 2008) of Python3 was then used to establish the metabolic network layer based on the following statements: (1) the proteins/enzymes, if having an ortholog in sugarcane, were set as the nodes of the graph for a practical and biological point of view and further data integration; (2) the connections among the nodes were given by undirected edges created when the product of any reaction associated with a protein was the substrate for any reaction connected with a subsequent protein; (3) if there was no protein listed to a reaction, the reaction was set as a node in the graph; and (4) if a certain protein was related to a reversible reaction, the product of such reaction was also considered as a substrate of that protein. Although product–substrates are direct relationships, the use of an undirected graph permits the modeling of the second PPI layer.

### Modeling of the PPI Layer

The PPI layer was modeled over the metabolic layer using both text mining and experimental data. Experimental data were obtained for *A. thaliana* from the BioGRID database. Nodes were set as either new or merged with the existing nodes already established in the first layer of the network when parsing the SwissProt text mining and BioGRID interactions data, still only considering those nodes having predicted sugarcane orthologs. In the latter case, the merge was performed by updating all node attributes. Connections among nodes were followed as undirected edges of PPI networks. The full assembled network is provided as a GRAPHML file on Mendeley Data (doi: 10.17632/5vtg4rk89j.1).

### Network Analysis and Statistics

We further investigated the sugarcane dual-layered network using the NetworkX package of Python3. We ranked and classified the nodes according to their measures of centrality in the network by using an unsupervised learning *K*-means clustering algorithm. Nodes with higher values of centrality measures, such as degree and betweenness centralities, may work as *hubs* since they interact with a large number of other nodes in the networks. Degree centrality is used to rank central nodes according to the number of connections with other nodes, whereas betweenness centrality ranks nodes based on the proportion of paths between two other nodes in the networks that the targeted node is involved in, unraveling the essential nodes for the communication between neighbor nodes. Other topology statistics such as density, ACC, and average shortest path length were also computed.

We also investigated the presence of densely connected modules using the Molecular Complex Detection (MCODE) algorithm (Bader and Hogue, 2003) wrapped into Cytoscape v3.8.1 (Shannon et al., 2003) with default parameters. Finally, we examined the putative signaling extension of the DE of RGAs found as DE. For this analysis, we used a DFS algorithm implemented in NetworkX to traverse the dual-layered network from nodes harboring RGAs found as differentially expressed (RGA-DEs) as the sources and across all subsequent nodes harboring differentially expressed genes (DEGs).

### Gene Set Enrichment Analysis

In an attempt to identify the most significant genes conferring the differences in the levels of smut resistance among the two targeted sugarcane genotypes of this study, we utilized the GSEA v4.1 software (Subramanian et al., 2005) to analyze gene sets GMT files obtained from densely connected module predictions with MCODE software. The expression input GCT file consisted of transcript count-per-million (CPM) values trimmed mean of *M*-values (TMM) normalized for all the control and inoculated biological replicates of each of the two transcriptome experiments (*N* = 12). No collapse parameter was set, and phenotypes were compared as inoculated vs. control within sugarcane experiments. GSEA calculates the gene set ES that represents the maximum deviation from zero and reflects the degree to which a gene set is over-represented. The minimum size and the maximum size of gene sets were set as 15 and 500, respectively. Statistical significance was calculated based on 1,000 gene set permutations. ESs for single gene sets were considered significant if the calculated nominal *p*-value < 0.05. GSEA leading-edge genes representing core members of high-scoring gene sets that contributed to the calculated significant NES were further functionally evaluated.

## Data Availability Statement

The original contributions presented in the study are included in the article/Supplementary Material, further inquiries can be directed to the corresponding author.

## Author Contributions

HR and CM-V conceived the study and designed the analysis. HR performed all networks and genomics analyses. HR, LR, and CM-V wrote the manuscript. SC, LC, M-AV, and LR provided expertise and editing. All authors reviewed and approved the manuscript.

## Conflict of Interest

The authors declare that the research was conducted in the absence of any commercial or financial relationships that could be construed as a potential conflict of interest.

## Publisher’s Note

All claims expressed in this article are solely those of the authors and do not necessarily represent those of their affiliated organizations, or those of the publisher, the editors and the reviewers. Any product that may be evaluated in this article, or claim that may be made by its manufacturer, is not guaranteed or endorsed by the publisher.

## Footnotes

1
https://www.arabidopsis.org/

2
https://www.uniprot.org/

3
https://www.kegg.jp/kegg/rest/
