# Supplementary material for: Arabidopsis-Based Dual-Layered Biological Network Analysis Elucidates Fully Modulated Pathways Related to Sugarcane Resistance on Biotrophic Pathogen Infection
Source: Front Plant Sci. 2021 Aug 19;12:707904. doi: 10.3389/fpls.2021.707904 (PMC8417329; doi:10.3389/fpls.2021.707904)
Supplement: Supplementary file 1 [file Data_Sheet_1.docx]

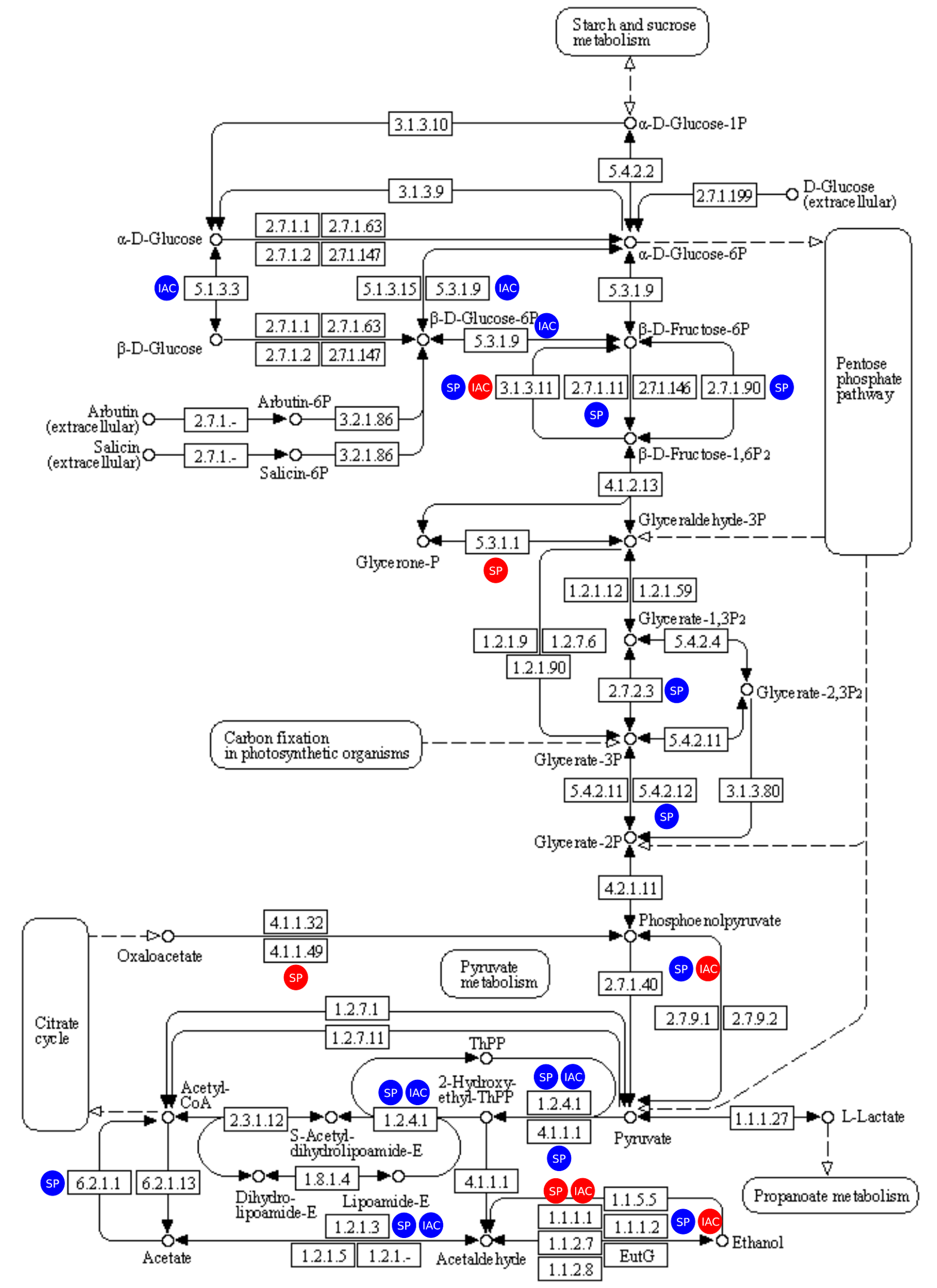


**Supplementary Figure 1**. Glycolysis pathway adapted from KEGG pathways database. Differential expressions of sugarcane orthologs are depicted as blue (down-regulation) or red (up-regulation) circles next to enzymes. Names within circles identify in which transcriptomes of IAC66-6 (IAC) and SP80-3280 (SP) the differential expression was predicted.
