# Supplementary material for: Arabidopsis-Based Dual-Layered Biological Network Analysis Elucidates Fully Modulated Pathways Related to Sugarcane Resistance on Biotrophic Pathogen Infection
Source: Front Plant Sci. 2021 Aug 19;12:707904. doi: 10.3389/fpls.2021.707904 (PMC8417329; doi:10.3389/fpls.2021.707904)
Supplement: Supplementary file 2 [file Data_Sheet_2.docx]

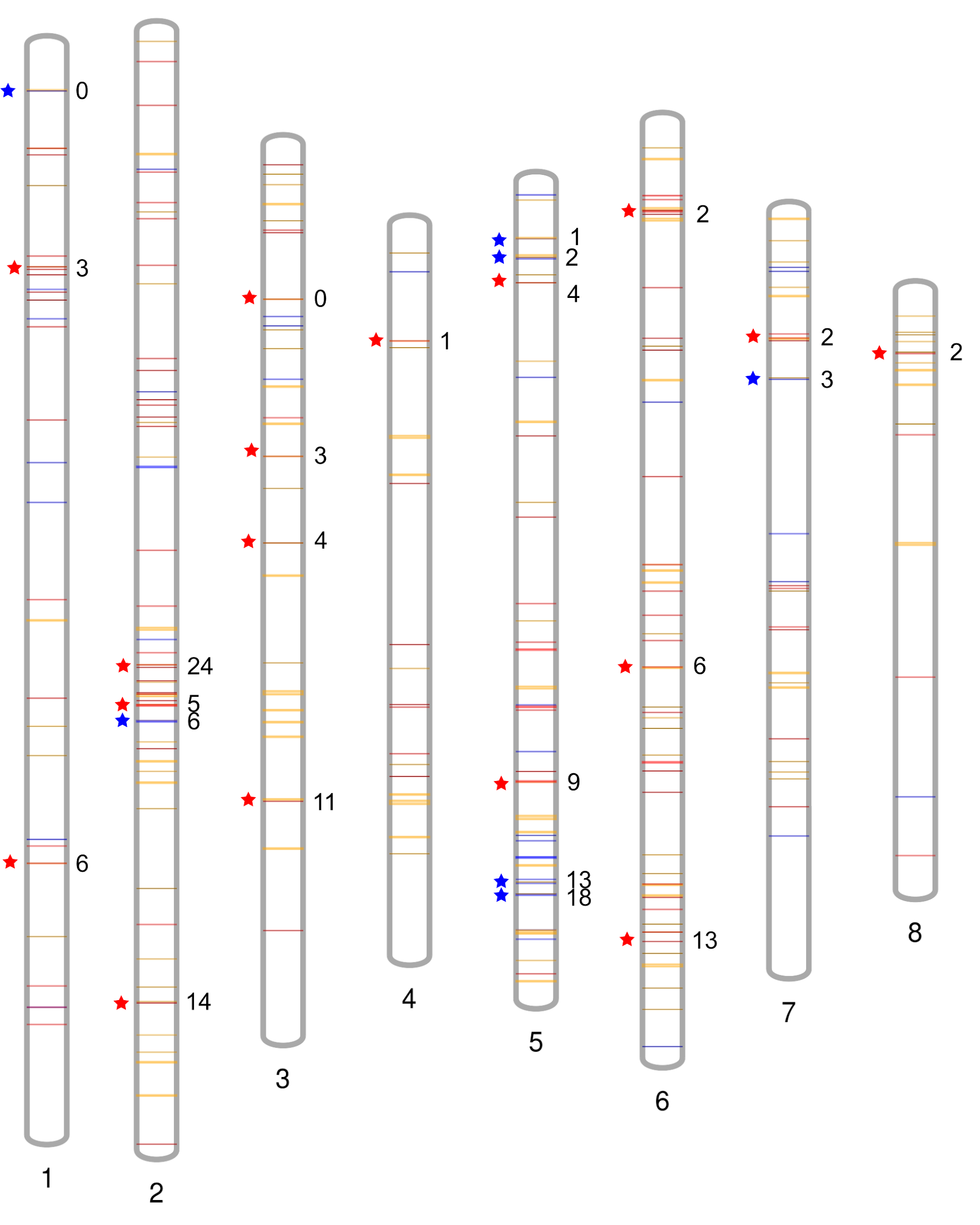


**Supplementary Figure 2.** Distribution along eight chromosomes of *Saccharum spontaneum* AP85-441 orthologs of RGA-DEs found as within core-periphery sub-networks originated exclusively from the experiments with sugarcane genotypes of IAC66-6 (blue lines) and SP80-3280 (red lines). Yellow lines depict RGA clusters identified by Rody et al 2019. Blue and red stars indicate RGA orthologs, from IAC or SP respectively, found within RGA clusters indicated by numbers in the right side of chromosomes. Ideogram figure was generated using the Phenogram web platform ([http://visualization.ritchielab.org/](http://visualization.ritchielab.org/phenograms)) (Wolfe et al 2013) and edited using InkScape Software.
