# Supplementary material for: Arabidopsis-Based Dual-Layered Biological Network Analysis Elucidates Fully Modulated Pathways Related to Sugarcane Resistance on Biotrophic Pathogen Infection
Source: Front Plant Sci. 2021 Aug 19;12:707904. doi: 10.3389/fpls.2021.707904 (PMC8417329; doi:10.3389/fpls.2021.707904)
Supplement: Supplementary file 3 [file Table_1.docx]

### **Supplementary Table 1.** Number of nodes predicted as cutting vertices within four K-means degree centrality groups (A-D).

| **Cutting vertices numbers** | **A** | **B** | **C** | **D** | **Total** |
| --- | --- | --- | --- | --- | --- |
| Nodes | 389 | 180 | 57 | 28 | 654 |
| RGA nodes | 7 | 22 | 8 | 0 | 37 |
| RGA nodes IAC DE | 3 | 3 | 1 | 0 | 7 |
| RGA nodes SP DE | 3 | 5 | 1 | 0 | 9 |
| Orthologs | 1,088 | 677 | 357 | 105 | 2,227 |
